# Preparation and Characterization of Poly(ethylene-*co*-vinyl alcohol)/poly(ε-caprolactone) Blend for Bioscaffolding Applications

**DOI:** 10.3390/ijms21165881

**Published:** 2020-08-16

**Authors:** Abdulaziz Ali Alghamdi, Hussain Alattas, Waseem Sharaf Saeed, Abdel-Basit Al-Odayni, Ali Alrahlah, Taieb Aouak

**Affiliations:** 1 Chemistry Department, College of Science, King Saud University, P.O. Box 2455, Riyadh 11451, Saudi Arabia; aalghamdia@ksu.edu.sa (A.A.A.); hussein_attas@hotmail.com (H.A.); 2 Engineer Abdullah Bugshan Research Chair for Dental and Oral Rehabilitation, College of Dentistry, King Saud University, Riyadh 11545, Saudi Arabia; aalodayni@ksu.edu.sa (A.-B.A.-O.); aalrahlah@ksu.edu.sa (A.A.); 3 Restorative Dental Sciences Department, College of Dentistry, King Saud University, Riyadh 11545, Saudi Arabia

**Keywords:** poly(ethylene-*co*-vinyl alcohol)/poly(ε-caprolactone) miscibility, thermal properties, mechanical properties, surface wettability, cell adhesion, pore interconnection

## Abstract

In order to improve the cell adhesion on poly(ε-caprolactone) (PCL) scaffolds, poly(ethylene-*co*-vinyl alcohol) (E-VAL) which has hydroxyl groups capable of developing hydrogen bonds with celling was blended with this polymer. To reach this goal, a series of E-VAL/PCL blends with different compositions were prepared by the solvent casting method. The miscibility of the polymer blend was proved by differential scanning calorimetry and Fourier-transform infrared spectroscopy spectrometry. Furthermore, the mechanical properties of the polymer blends were assessed in their wet state by dynamic mechanical analysis. The surfaces wettability of blends and their components were examined through static contact angle measurements. The pore interconnections in the resulted scaffolds were achieved by the incorporation of naphthalene microparticles which were used as porogen and then removed in its gas state by sublimation under reduced pressure. The presence of pores interconnected inside the polymeric materials and their surface morphologies was examined by scanning electron microscopy. The in-vitro cytotoxicity and cell adhesion on the prepared materials were examined by an MTT (3-(4,5-dimethylthiazol-2-yl)-2,5-diphenyltetrazolium bromide) assay.

## 1. Introduction

The development and commercialization of new polymers are costly efforts, which usually require many years of work. Combining two or more different polymers has now become a compelling alternative to easily obtain new polymeric materials with desirable properties. However, most blends of high-molecular-weight polymers are immiscible [1,2,3,4]. That is, when polymers are mixed, they form separate phases. This characteristic, combined with low physical attraction forces across phase boundaries, usually causes immiscible blend systems with poor mechanical properties. Poly(ε-caprolactone) (PCL) also called Shapelok (US) or Polymorph (UK) is a nonhazardous polymer that can be easily shaped by hand due to its low melting point (60 °C). One of the principal routes employed to obtain this polymer is the ring-opening polymerization of ε-caprolactone using stannous octoate as a catalyst [5]. The competitive viscoelastic and rheological properties of PCL enormously facilitate their manufacture and handling into a large range of implants and other devices [6]. In addition, it is a food and drug administration (FDA)-approved biodegradable and biocompatible polymer, and with the availability of low-cost production routes, it provides a promising base for producing long-term degradable implants since it can be physiochemically tailored to control the biodegradation process to suit specific anatomical needs [7]. However, PCL does not provide a desirable environment for cell adhesion and growth due to the lack of biological recognition sites on its surface, which could reduce its applicability in bioscaffolds [8,9,10]. Plasma treatment allows the incorporation of different functional groups on the surface of PCL, thereby tuning its properties [5,6]. This route also permits the modification of the surface energy and surface wettability of the resulting polymer, leading to drastic changes of the cell adhesion behavior [5,6,7]. Different authors reported that PCL offers competitive candidacy when used in tissue engineering applications due to its biocompatibility, biodegradability, high stability, and formability [11,12,13,14,15]. The results obtained revealed significant enhancement of cell adhesion and proliferation. The modification of the surface of PCL with the cell attractive peptide derivative Arg-Gly-Asp-Cys (RGDC) using the chemical immobilization procedure was investigated by Zhang and Hollister [12]. The results showed that the PCL with RGDC modification promoted initial bone marrow stromal cell (BMSC) attachment, spreading, and formation of focal adhesion. The cell adhesion, tensile strength, and miscibility of PCL blended with poly(L-lactic acid) (PL-LA) was investigated by Khatri et al. [16] A comparative enhancement of cell adhesion was found for PL-LA and PCL/PL-LA blends containing higher PL-LA content. They showed that the specimens with less PL-LA content showed higher tensile strength. The suitability of PCL/PL-LA nanofibrouse tubes was demonstrated for nerve tissue regeneration and tissue growth.

Poly(ethylene-co-vinyl alcohol) (E-VAL) is a copolymer containing controllable hydrophilic and hydrophobic properties due to its comonomer composition. The presence of hydroxyl groups promotes cell adhesion due to the hydrophilic character of the cells [17]. This copolymer is synthesized by the polymerization of ethylene with vinyl acetate giving the poly(ethylene-co-vinyl acetate) following the hydrolysis of functional groups. E-VAL is also biodegradable [17], biocompatible [18,19,20,21], and possesses excellent mechanical and gas barrier properties. Few investigations have been reported on the application of this copolymer in the biomedical domain. Matsumura et al. [20] demonstrated the control of the differentiation and proliferation of the periodontal cells used to develop a highly organized hybrid implant. Hassanzadeh et al. [21] reported on the surface modification of E-VAL by succinylation to obtain succinate modified E-VAL (EVOHS) to improve the structural properties and evaluated its ability to deliver epirubicin to hepatocellular carcinoma cells in response to varying temperature. They revealed that all the characteristic properties improved as the hydrophilicity of micelles increased, and these entities can be effective as thermo responsive delivery systems for targeting the delivery of cytotoxic agents to hepatocellular carcinoma. The use of biocompatible and biodegradable porous scaffolds as 3D models has been widely investigated in vitro and in vivo. Highly porous scaffolds allow cells to spread and facilitate their interaction with biological liquid vessels, supply nutrients to the transplanted cells, and eliminate waste products. The high porosity of scaffolds is needed to reproduce adequate tissues. The biochemical properties and geometric structure of such scaffolds should support cell attachment, migration, growth, and ultimately, tissue maturation [22]. For tissue reproduction, the density and specific surface area of the pores must be high enough so that they can form fully interconnected 3D structures with good structural and mechanical properties [23,24,25]. Solvent casting, freeze-drying, phase inversion, fiber bonding, melt-based technologies, and high pressure-based methods, have been used to prepare polymeric scaffolds [26], and some of these methods generated randomly structured constructs with unpredictable pore sizes and connections. In the solvent casting technique, porous polymers are prepared by solvent evaporation from a suspension containing solid particles called porogen and polymeric solution, and removing porogen from the polymer matrix. Among the many porogens used in this domain are sodium chloride, ammonium bicarbonate, and glucose with different crystal sizes [27,28,29,30,31].

In this work, our aim was (i) to enhance cell adhesion to PCL and improve its mechanical properties by blending it with E-VAL and (ii) create an interconnected porous construct as a bioscaffold. To reach this goal, a series of E-VAL/PCL blends were prepared by solvent casting, and their miscibility was examined by differential scanning calorimetry (DSC), Fourier-transform infrared spectroscopy (FTIR), X-ray diffraction (XRD), and scanning electron microscope (SEM) techniques. The mechanical properties of resulted materials were performed in their wet state by dynamic mechanical analysis and their surface wettability was examined through static contact angle measurements. The cell adhesion was performed by the colorimetric detection of bound cells based on the Kueng method and confirmed by phase-contrast microscopy to measure the flattening of adherent cells using the Yamada and Kennedy method [32]. The interconnected pores were produced by the naphthalene (Naph) microparticles used as porogens. The interconnection of pores was examined for the selected miscible materials.

## 2. Results and Discussion

### 2.1. Miscibility

#### 2.1.1. Preliminary Test

Preliminary tests carried out on the miscibility of the E-VAL/PCL blends revealed that their blends are miscible in the composition range investigated. Indeed, the presence of a single limpid stable phase of their solutions in dimethylformamide (DMF) and a homogeneous film devoid of any heterogeneous zones obtained after solvent evaporation, as observed with an optical microscope, confirm their miscibility.

#### 2.1.2. FTIR Analysis

**Figure 1:**
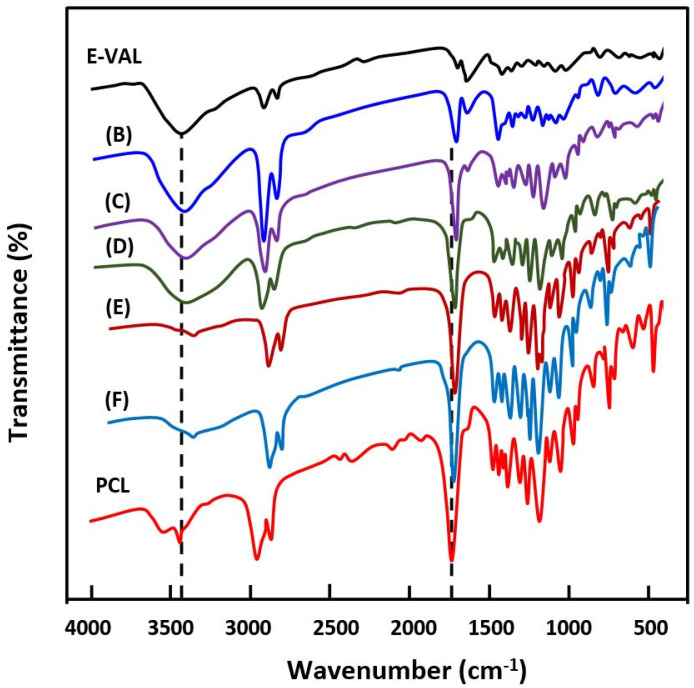
FTIR spectra of poly(ethylene-*co*-vinyl alcohol) (E-VAL), poly(ε-caprolactone) (PCL) and their blends containing (B) 90, (C) 75, (D) 50, (E) 25 and (F) 10 wt% E-VAL.

**Scheme 1:**
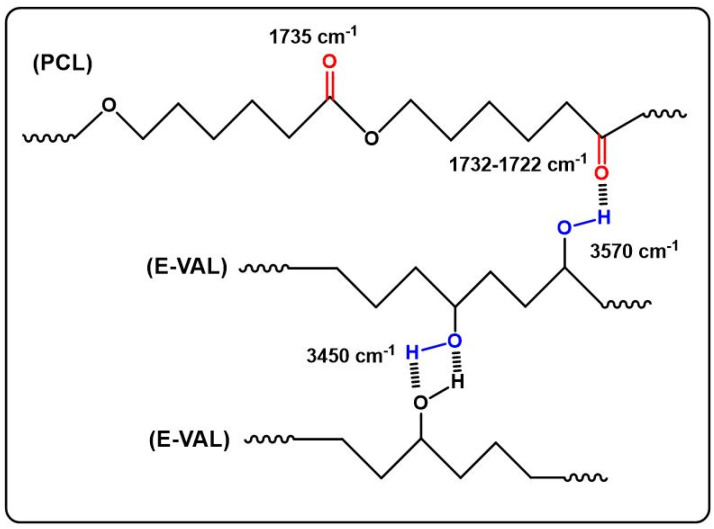
Hydrogen bonds in the E-VAL/PCL blends.

**Figure 2:**
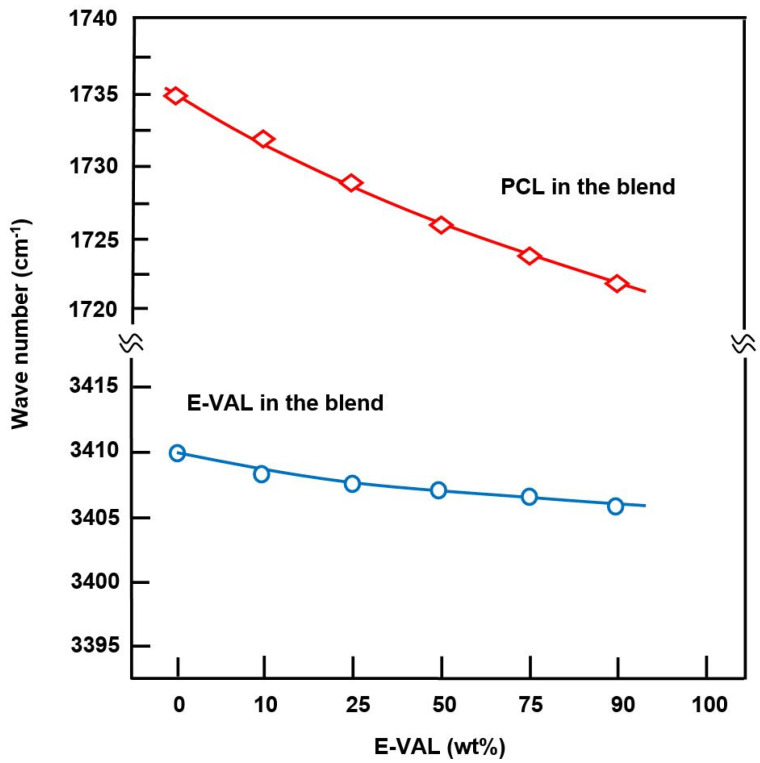
Shift variation in the absorption bands of the hydroxyl groups of E-VAL and the carbonyl groups of PCL in E-VAL/PCL blends versus E-VAL content.

The FTIR spectra of E-VAL/PCL blends and their components are shown in Figure 1. The absorption bands at 3410 cm^−1^ and 1725 cm^−1^ correspond to the hydroxyl group of the alcoholic unit of the copolymer and the carbonyl group of the homopolymer, respectively. In general, these bands slightly shifted toward the lower wavenumber when the E-VAL content increased in the blend. The profile of the changes in the wavenumber of two characteristics absorption bands versus the E-VAL content in the blend are presented in Figure 2. According to different authors [33,34,35], the shift or broadening of the absorption band or both reflect the presence of hydrogen bonds between the different components of the blend, leading to its miscibility. Indeed, E-VAL/PCL spectra show these characteristics, reflecting the presence of hydrogen bonds between the hydroxyl groups of E-VAL vinylic units and the carbonyl of ε-caprolactone units, as shown in Scheme 1. When the chains of PCL are diluted using excess E-VAL, the hydrogen of the hydroxyl group interacts with the oxygen of the PCL carbonyl group.

**Figure 3:**
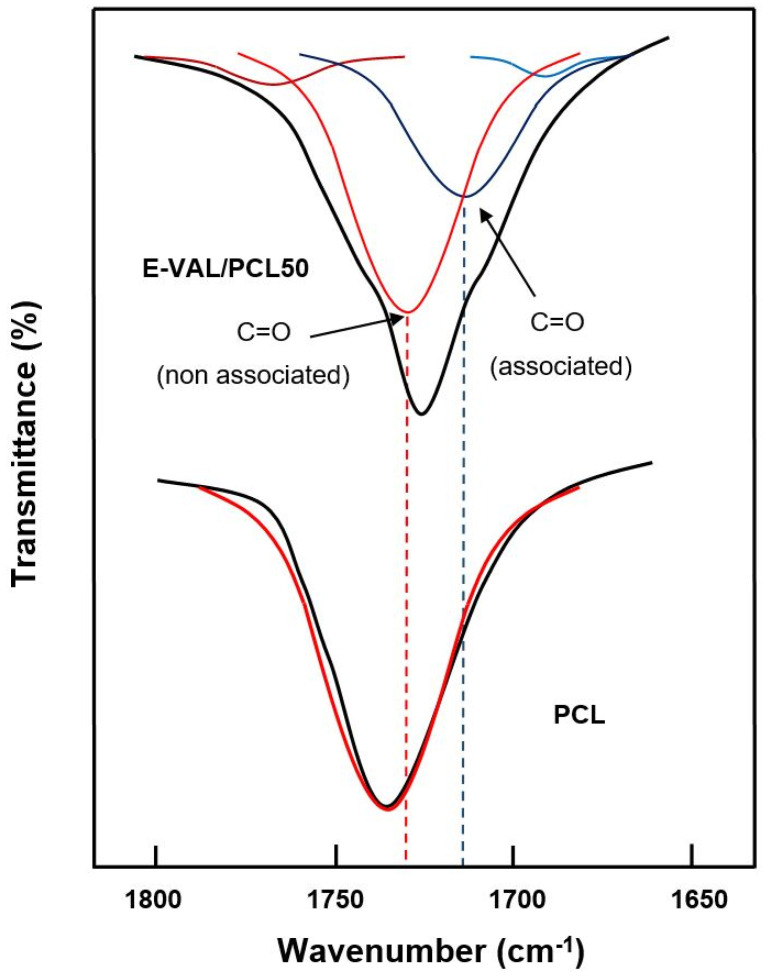
Deconvolution of the absorption band between 1700 and 1800 cm^−1^ in free and associated carbonyl peaks of pure E-VAL and E-VAL/PCL50 blend.

The data processing of the absorption bands localized between 1700 and 1800 cm^−1^ revealed two principals peaks assigned to the free and associated carbonyls, as shown in Figure 3. The fractions of the associated carbonyl bonds were evaluated by Equation (1) [36].

(1) $$F_{a}=1-\frac{A_{na}}{A_{na}+A_{a}\times \varepsilon }$$

where *A_a_* and *A_na_* are the relative areas corresponding to the associated and free carbonyl bonds, respectively. *ε* is the absorptivity fraction of these two bonds. According to the literature, for the carbonyl group, *ε* is equal to 1.1 [37]. The fractions of the associated and free carbonyls were calculated from *A_a_* and *A_na_* and Equation (1), and the results obtained are listed in Table 1.

**Figure 4:**
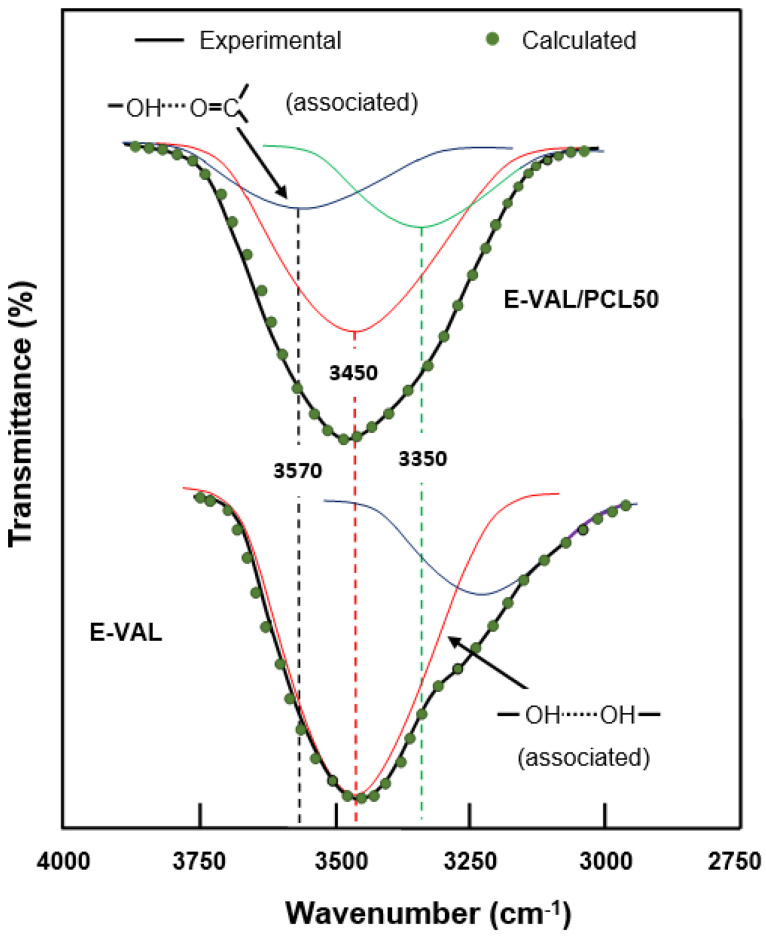
Deconvolution of the absorption band between 3000 and 3750 cm^−1^ in free and associated hydroxyl peaks of pure E-VAL and the E-VAL/PCL50 blend.

On the hydroxyl side, the situation seems to be more complex due to the overlap of several absorption bands resulting from the vibration of free hydrogen groups belonging to the E-VAL and those of the associated inter- and intra-chains of this copolymer. The presence of hydroxyl and carbonyl groups in the blend can generate hydroxyl–hydroxyl (intra-chain), hydroxyl–hydroxyl (inter-chains), and hydroxyl–carbonyl hydrogen bonds leading to three absorption bands. The presence of traces of water molecules incrusted in the E-VAL matrix can also develop hydrogen bonds with the components of the blend resulting in a shift, or a broad band, or both. We tried to find these absorption bands by the deconvolution of pure E-VAL and E-VAL/PCL spectra between 3000 and 3750 cm^−1^, as shown in Figure 4. As can be seen from the two spectra, the absorption band characterizing the O–H stretching of E-VAL vinylic units reveals several Lorentzian peaks between 3000 and 3750 cm^−1^. The signal localized at 3450 cm^−1^ is attributed to the O–H H–O associated group of the vinylic units [38] and that observed in the blend system at 3570 cm^−1^ reflects the new distribution of hydrogen bonds between the hydroxyl–hydroxyl and the hydroxyl–carbonyl interactions.

#### 2.1.3. DSC Analysis

##### Glass Transition Temperature

**Figure 5:**
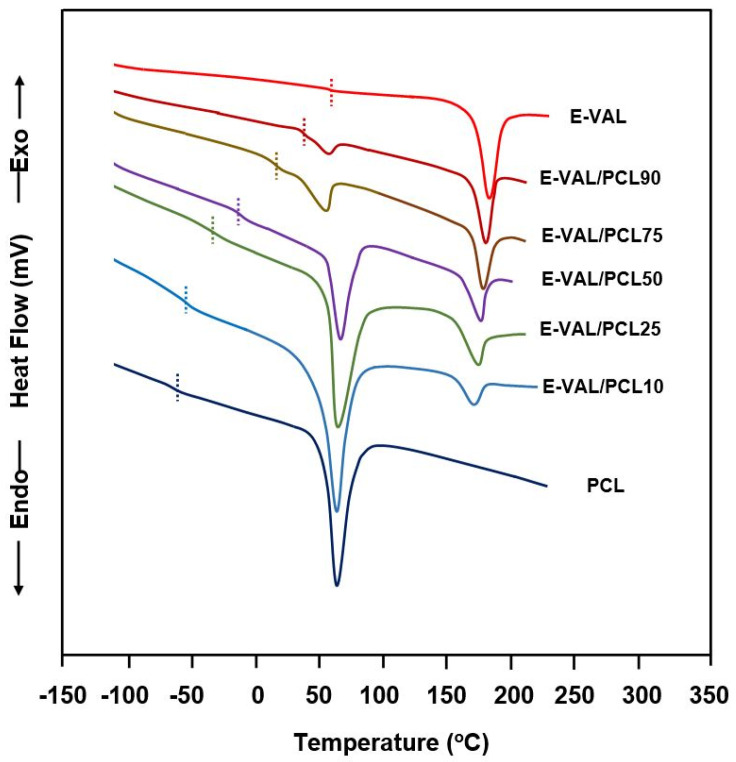
Heating differential scanning calorimetry thermograms of E-VAL/PCL blends and their pure components.

The DSC thermograms of E-VAL/PCL blends and their pure components are shown in Figure 5, and the corresponding glass transition temperatures are grouped in Table 2. The presence of only one transition temperature localized between their pure components confirms the miscible behavior of the polymer blend in their amorphous region. The *T_g_* value of PCL shifted toward higher temperatures when the E-VAL content in the blend increased. According to Qui et al. [39], the appearance of a single *T_g_* for a blend indicates full miscibility at a 20–40 nm scale.

**Figure 6:**
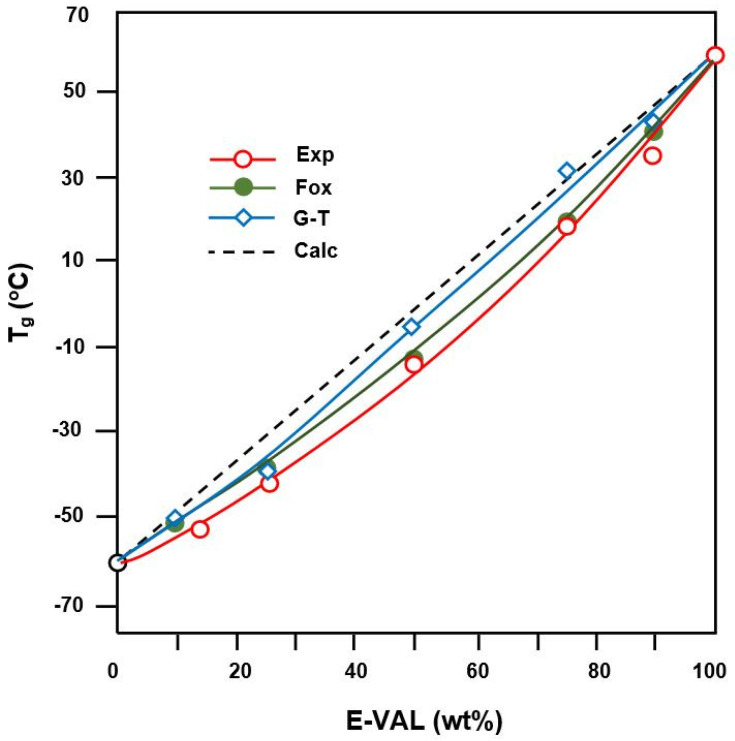
Comparison of *T_g_s* of E-VAL/PCL blends with different compositions determined by experiment, calculation, and using the Fox equation and Gordon–Taylor equation.

The experimental glass transition temperatures, $T_{g}^{Exp}$*s* values were deduced from the E-VAL/PCL thermal curves in Figure 5. The comparison of these values with those calculated from the average arithmetic of pure components, $T_{g}^{cal}$, according to Equation (2) is shown in Table 2. According to different authors [40,41,42], a negative deviation from the ideality characterized by the $T_{g}^{cal}$-composition linear curve indicates a miscible blend.

(2) $$T_{gm}^{Calc}\left(\mathrm{blend}\right)=\frac{w_{1}T_{g}{}_{1}+w_{2}T_{g}{}_{2}}{w_{1}+w_{2}}$$

where $T_{g1}$ and $w_{1}$ are the glass transition temperature of pure E-VAL and its weight fraction, respectively, and $T_{g2}$ and *w*_2_ are the glass transition temperature of PCL and its weight fraction, respectively. The shift in $T_{g}^{Exp}$ from that linearity indicates the presence of specific interactions between the two polymers. Different approaches based on the experimental $T_{g}$*s* of the pure components have been suggested to predict the profile of the $T_{g}$ change in a miscible blend versus its composition. Among the most common approaches, the Fox (Equation (3)) [43] and Gordon–Taylor (Equation (4)) [44] were selected to study the *T_g_* behavior of the polymer blends.

(3) $$\frac{1}{T_{g}{}_{blend}}=\frac{w_{1}}{T_{g}{}_{1}}+\frac{w_{2}}{T_{g}{}_{2}}$$

(4) $$T_{g}{}_{Blend}=\frac{w_{1}T_{g}{}_{1}+k(1-w_{1})T_{g}{}_{2}}{w_{1}+k(1-w_{1})}$$

(5) $$k=\frac{\Delta \alpha_{1}}{\Delta \alpha_{2}}$$

where *k* is an adjustable fitting parameter in the Gordon–Taylor equation, and Δ*α_i_* is the change in the expansion coefficient at *T_gi_*. As can be seen from the data presented in Table 2 and plotted in Figure 6, the *T_g_* values of the blends fit reasonably well with those calculated using the Fox equation and Gordon–Taylor equations at *k* = 1.5 and are slightly deviated from those calculated from the arithmetic mean, $T_{g}^{Cal}$ (blend). This behavior indicates the miscibility of this pair of polymer in all ratios. In addition, the change in the expansion of the E-VAL macromolecules increased 1.5 fold compared with that of PCL, thus revealing a good deployment of the copolymer chains and in turn, the miscibility characteristic of this binary system.

##### Melting Temperature

The DSC curves indicating the melting points of E-VAL, PCL and their blends with different compositions are shown in Figure 5 and the data of the apparent melting temperature and the heat of melting deduced are listed in Table 3. Two distinct endothermic sharp peaks attributed to the melting points of each pure component were observed in the E-VAL/PCL thermal curves. As shown in these thermal curves, the *T_m_* value of PCL in the blend moderately shifted toward low temperatures (from 183 °C to 167 °C); at the same time, the *T_m_* of PCL demonstrated a maximum value of 67 °C when the composition was 50 wt%.

In the case of a miscible polymer blend, a decrease in the melting temperature of a semicrystalline polymer in a blend is due to the thermodynamic interactions between the different types of polymer chains. According to the Flory–Huggins theory developed by Nischi and Wang [46], a miscible blend is characterized by the magnitude of the decrease in the melting temperature, which makes it possible to measure the interaction energy. The melting temperature of a polymer is generally affected by thermodynamic factors and morphological parameters, in particular, the thickness of the crystal.

### 2.2. Mechanical Properties

**Figure 7:**
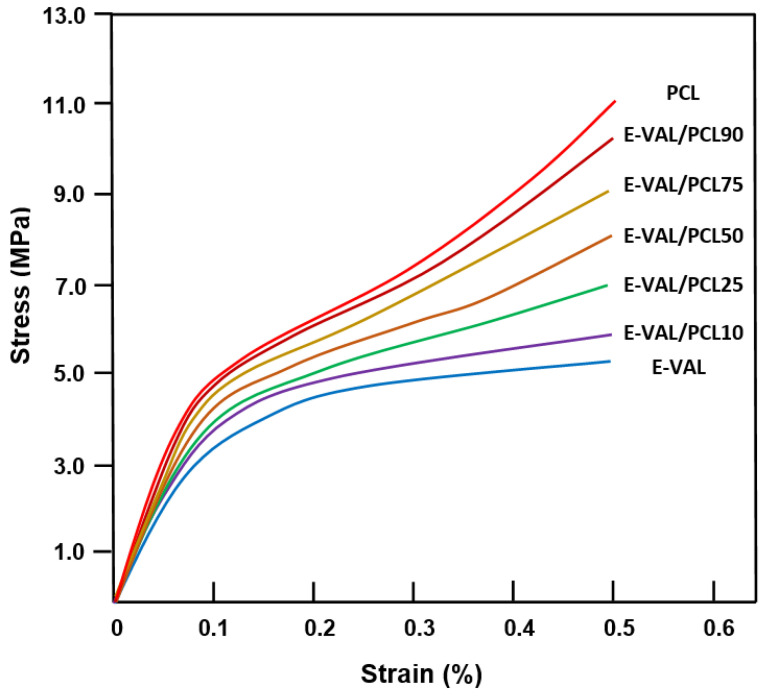
Stress–strain curves of wet PCL, E-VAL and E-VAL/PCL scaffolds with different E-VAL contents.

The effect of E-VAL incorporated in the PCL matrix on the mechanical performance of the fabricated scaffold was studied in their wet state through compressive mechanical tests, and the results obtained are shown in the compressive stress–strain curves in Figure 7. As can be seen from these curve profiles, all these materials present a typical stress–strain pattern that is specific to a highly porous scaffold, as observed by others [47,48]. The profiles of these curves show three different zones: the first zone is practically linear which was observed at weak strain (0.05–0.12) indicating a rigid mechanical response associated with an elastic behavior of materials, the second zone is characterized by a pseudo-plateau suggesting a region with lower stiffness, and the last zone called “the densification region” is characterized by an increase in the stress with the increasing strain linked to the densification of porous materials [47,48,49,50,51]. According to the report published by Nicoll [52], the compression strength of the cancellous and cortical bone tissues varies depending on their bone densities between 5 and 50 MPa and 130 and 180 MPa, respectively. Others reported that the mechanical properties of bone depend on its structure, orientation, and water content and the values of the compression modulus of normal wet human cancellous bone vary between 12 and 900 MPa [53]. Although the mechanical behavior of scaffolds was usually reported in their dry state, the evaluation of their mechanical properties in the hydrated state is physiologically fairer. The results of the compressive modulus, *E*, and maximum stress, *σ_max_*, of wet PCL, wet E-VAL and wet E-VAL/PCL scaffolds are shown for comparison in Table 4. For wet PCL, the *E* and *σ_max_* values were 66.67 ± 1.23 and 11.06 ± 1.11 MPa, respectively, in which the first value is greater than 18.37% of that reported, and the second is in agreement with [54] (54.42 ± 2.42 MPa, 10.96 ± 0.92 MPa). Indeed, these results are consistent because PCL as a hydrophobic polymer does not absorb the aqueous solution; therefore, its behavior with water does not cause a noticeable change in its mechanical properties, while E-VAL, due to its high affinity for the same medium, demonstrates remarkable wetting and modification in its properties. The *E* and *σ_max_* values of this scaffold in its wet state were 37.50 ± 1.56 MPa and 5.25 ± 0.44 MPa, respectively, and for its dry state, the E value taken from the database in the literature ranged between 61.1 and 85.5 MPa [55]. Concerning the scaffolds of the blends, the values of these two parameters drop practically by half when the E-VAL incorporated in the blend goes from zero to 100% by weight. As can be expected, these values are included in the reported interval of the cancellous bone, which is significantly lower than that of cortical bone. Loading E-VAL in the PCL matrix, even in small amounts, affects the scaffold mechanical response, thus enhancing the mechanical properties of the scaffold. According to Thadavirul et al. [56], the decrease in the compressive modulus after immersion in aqueous solution is attributed to the plasticization effect due to the water absorbed, making it more flexible.

### 2.3. Surface Wettability

**Figure 8:**
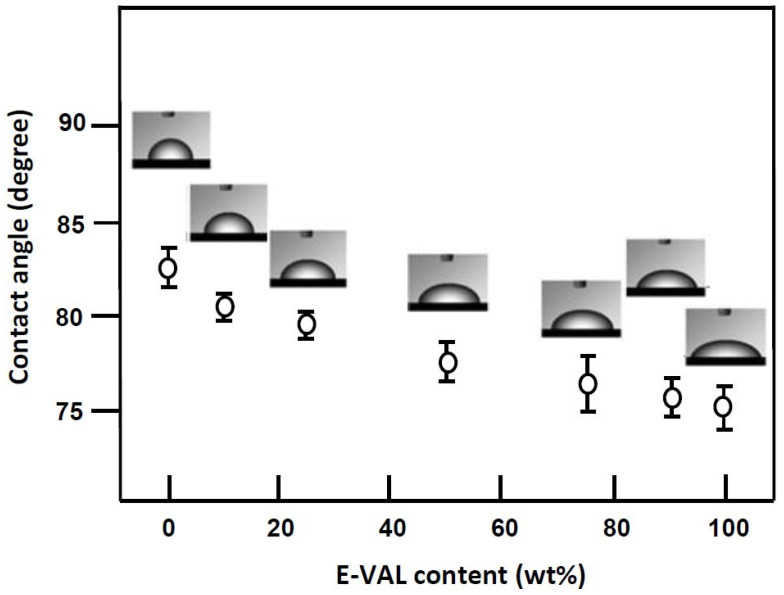
Variation of the static water contact angle of E-VAL/PCL blend versus the E-VAL content.

The wettability of the scaffold surfaces plays a key role in cell adhesion and growth. A higher wettability is associated with a higher surface energy, implying a lower contact angle [57]. The values of the contact angles measured for PCL, E-VAL and E-VAL/PCL films with different E-VAL content are shown in Figure 8. For pure PCL, the surface had a higher (*p* < 0.0001) contact angle of 82.5°, which agrees with that of the literature, indicating the hydrophobic character of this polymer [58]. On the other hand, with a contact angle of 75.06°, E-VAL, which has dense hydroxyl groups, shows a higher hydrophilicity than PCL. The incorporation of E-VAL in the PCL matrix significantly increased the wettability of the resulting material. The addition of 10 wt% of E-VAL improved the wetting degree by 2.27% and 25 wt% permitted the enhancement of the PCL wettability by 9.33%. Although these values seem to be relatively low, from a cytological point of view they are sufficient to allow cell adhesion and growth on the material.

### 2.4. Assessment of Cell Adhesion and Growth

**Figure 9:**
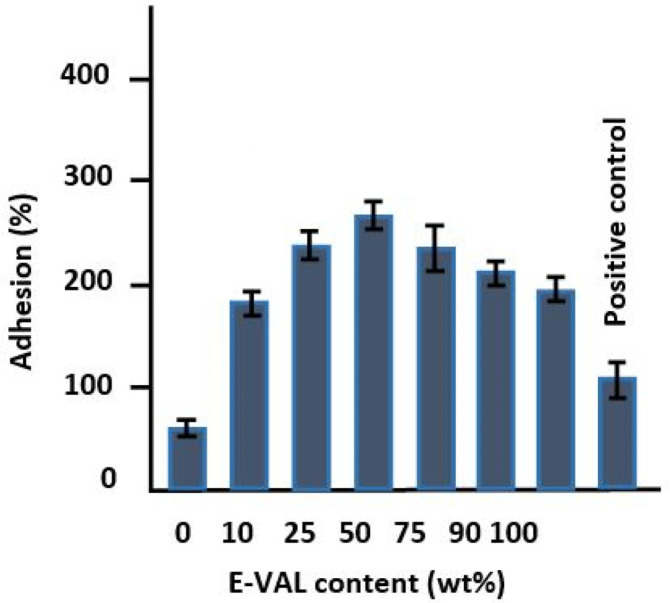
Cell adhesion tests on the prepared pure PCL, E-VAL and E-VAL/PCL materials with different E-VAL contents.

The results of the cell adhesion tests on the prepared polymeric materials are shown in Figure 9. The incorporation of E-VAL in the PCL matrix significantly improved the adhesion capability of LoVo cells onto PCL. An addition of only 10 wt% E-VAL increased the adhesion performance from 62.18 to 183.32% and the performance continued to increase up to 270% with E-VAL content. The improvement in the adhesion capability of LoVo cells on the PCL modified is probably due to two principal factors: an increase in its physicochemical affinity due to the hydrogen bonds developed and, the porosity enrichment on the surface of the resulting material when the hydroxyl groups carried by the E-VAL were added to this polymer. When the E-VAL content in the blend was more than 50 wt%, the decrease in adhesion performance is due to the reduction in pore size on the surface of the resulting material caused by excessive attraction forces created by an increase in the number of hydroxyl–hydroxyl hydrogen bonds of the excess of vinylic monomeric units of E-VAL in the blend. Therefore, the increased affinity is due to the hydrogen bonds between cells and the material through hydrogen bonds and the porosity enrichment on the surface. These factors can be considered important criteria to deploy this miscible blend as a scaffold material to obtain better cell adhesion and growth properties.

**Figure 10:**
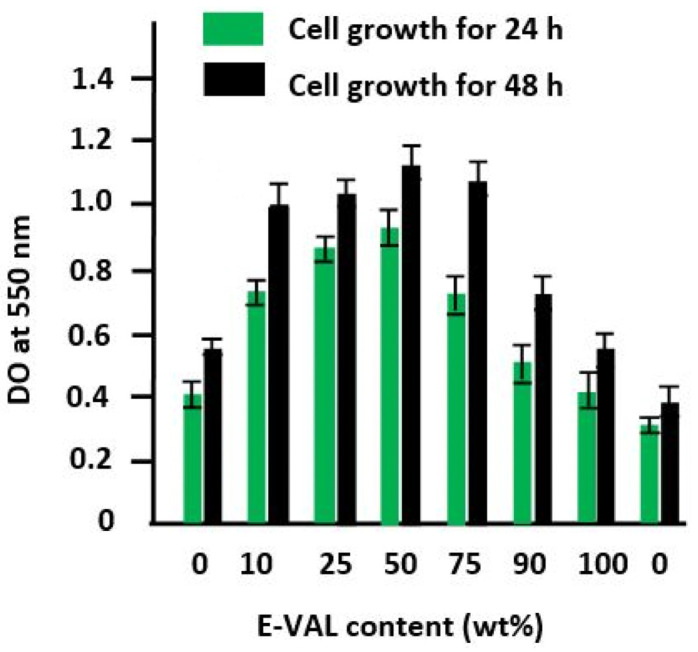
Cell growth tests on the pure PCL, pure E-VAL, and E-VAL/PCL materials with different E-VAL contents for 24 and 48 h.

In Figure 10, the diagram shows an increase in the growth rate of the cells on the surfaces of these materials following a similar trend as that of the cell adhesion shown in Figure 9. According to Abdelwafa et al. [11], the presence of hydroxyl or carboxyl groups in a scaffold promotes cell proliferation in a protein-mediated cell adhesion process.

### 2.5. Pore Morphology

#### 2.5.1. Pore Interconnection

**Figure 11:**
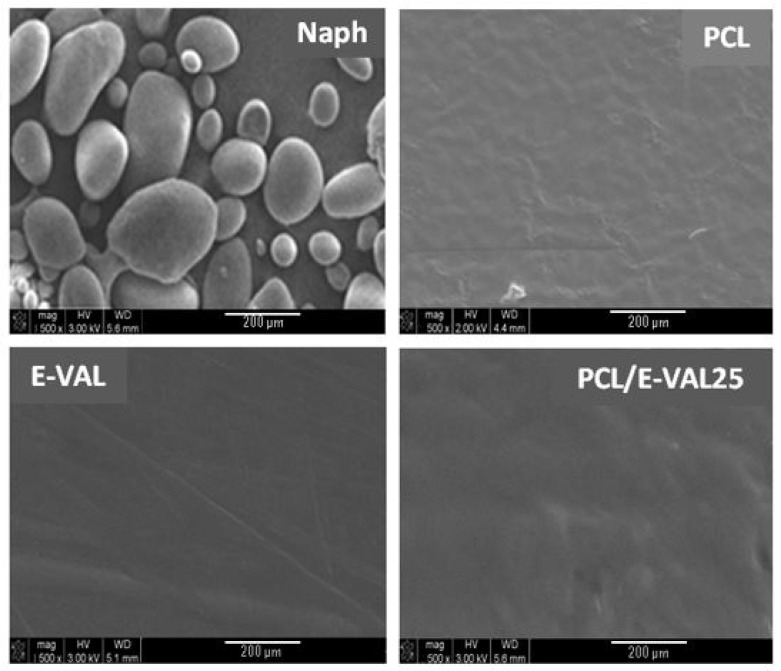
SEM micrographs of Naph microparticles and surface morphologies of PCL, E-VAL and PCL/E-VOH25 films.

**Figure 12:**
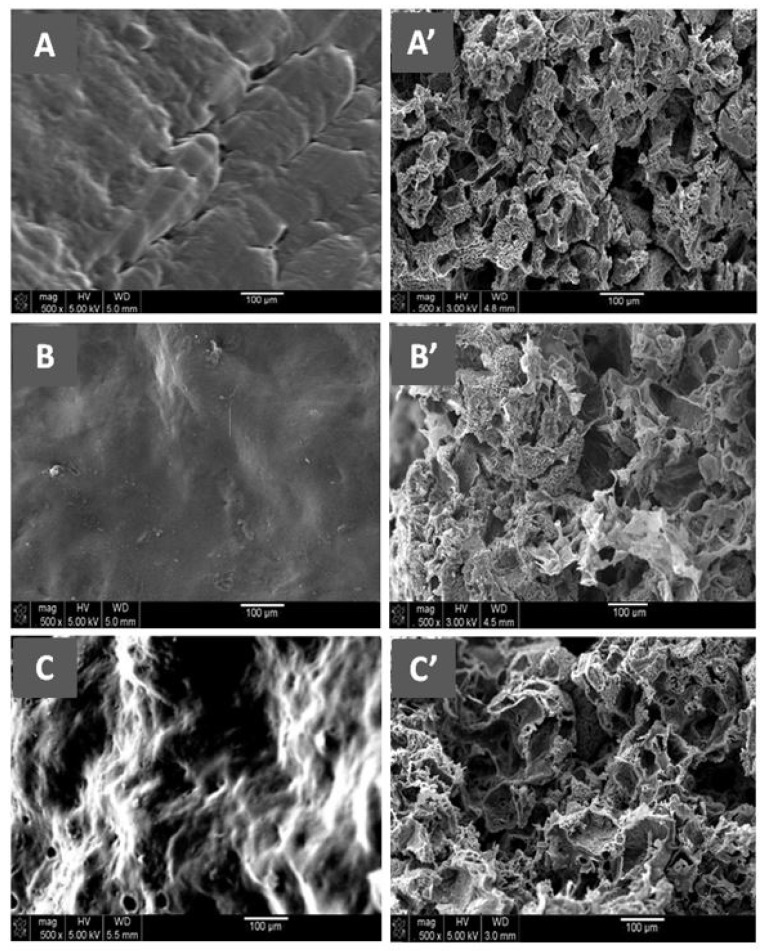
(Left) SEM micrographs of the surface morphologies of Naph microparticles embedded with (**A**) E-VAL, (**B**) PCL and (**C**) E-VAL/PCL25. (Right) the cross section morphologies of (**A’**) E-VAL, (**B’**) PCL and (**C’**) E-VAL/PCL25 after removing Naph microparticles.

Figure 11 shows the SEM micrographs of Naph microparticles and the surface morphologies of PCL, E-VAL, and E-VAL/PCL25 films taken before pore formation. The image of Naph microparticles shows, in general, oval forms whose dimensions vary between 80 *×* 70 × 70 and 250 *×* 100 *×* 100 μm^3^. The images of PCL, E-VAL, and PCL/E-VAL25 reveal practically similar surface morphologies, which are smooth and homogeneous, devoid of any relief which could be produced during sample preparation. In the case of E-VAL/PCL, such a surface is considered as another proof of miscibility. Figure 12 (left) shows the images of PCL, E-VAL, and E-VAL/PCL25 after porogen microparticle incorporation. The surface morphologies of the resulting materials are different from those before porogen incorporation, resembling mud covering a vegetal ground. These figures let us imagine the total coverage of the porogen by copolymer (A), copolymer (B), or their blends (C). The micrographs of Figure 12 (right) present the cross sections of the same samples after the removal of porogens. As can be observed, the inner morphology of the materials has a spongy form containing interconnected pores, which have heterogeneous dimensions similar to those of the porogen. The internal pore surfaces appear large enough to allow cells to proliferate quickly [59].

#### 2.5.2. Pore Diameters and Porosities

Porosity and pore size are important parameters of scaffolds, playing significant roles in cell proliferation and growth. According to Han et al. [60], to a certain limit, higher porosities and pores sizes favor the creation of a spacious environment that facilitates cell proliferation. At too high pore sizes and porosities exceeding 93%, it will be very difficult to preserve the mechanical rigidity of the scaffold at an acceptable level [60]. According to the literature, porosities of around 90% and pores sizes between 90 and 250 μm are reasonable for tissue engineering scaffolds [61,62]. In this investigation, the effects of the porogen microparticle diameters on the pore diameters and porosities of the prepared scaffolds were carried out using light microscopy, and the results are summarized in Table 5. These data revealed that the size of the pores formed is much smaller than expected due to the dissolution of a part of the porogen during suspension preparation containing naphthalene microparticles in viscous polymeric solution in DMF, and the contraction of the pores under the effect of the vacuum used to extract the porogen from these polymeric materials, especially at temperatures close to *T_g_*. To obtain homogeneous pores having the shape and size of pores similar to those of the pore-forming agent, this requires the use of slightly larger particles of homogeneous size, and their removal must be carried out under optimal temperatures and reduced pressures.

## 3. Materials and Methods

### 3.1. Materials

E-VAL (27% ethylene), PCL (${\bar{M}}_{w}$ = 45,000 g·mol^−1^), and Naph beads (purity, >99%) were provided from Sigma-Aldrich (Hamburg, Germany). DMF (purity, 98%) was purchased from Panreac Química SLU, Castellar del Vallès, Spain. All chemicals were used without prior purification.

### 3.2. Blend Preparation

E-VAL/PCL blend was prepared by solution casting. PCL and E-VAL were separately dissolved at 80 °C in DMF in a 50 mL Erlenmeyer flask under stirring until complete dissolution and formation of a viscous solution. The two prepared solutions were then mixed under continuous stirring to form an E-VAL/PCL/DMF ternary solution. To obtain a film sample of perfectly homogeneous thickness, the solution was poured into a Teflon Petri dish and then deposited horizontally on a square Styrofoam plate floating on the surface of a crystallizer filled with water and dried at ambient temperature for 48 h. To extract the residual solvent crusted in the obtained film, the Teflon plate containing polymeric material was then transferred to dry in a vacuum oven at 60 °C for 24 h. A series of E-VAL/PCL blends containing 10, 25, 50, 75, and 90 wt% E-VAL were prepared by the same method, and the experimental conditions are listed in Table 6.

### 3.3. Interconnected Pore Formation

Porogen microparticles were prepared by grinding Naph beads using a coffee grinder and then sieving through a series of sieves with pores sizes between 122 and 192 μm. The average diameter of the prepared Naph microparticles was estimated as 183 μm by SEM. A defined amount of Naph microparticles was immersed in a glass cylindrical capsule containing very viscous polymeric solution obtained after solvent evaporation at one-third of the total volume and maintained at −5 °C. The pore formation and interconnection were produced by removing porogens from the polymeric material according to the procedure described in the literature [63]. Removing Naph microparticles was done by sublimation and then extraction under reduced pressure at a temperature slightly lower than the glass transition temperature of the polymeric material. Note that a temperature slightly lower than *T_g_* makes the polymer softer, which makes it easier to destroy the walls separating the pores when high pressure difference is exerted on the walls.

### 3.4. Characterization

#### 3.4.1. Blend Miscibility

PCL, E-VAL and their blends were characterized by different techniques. FTIR analysis was recorded on a Nicolet 6700 FT-IR spectrometer (Thermo Scientific, Waltham, MA, USA). The spectra were obtained over a region of 4000–500 cm^−1^ at room temperature and acquired with a total of 32 scans per spectrum and a resolution of 2 cm^−1^. For qualitative and quantitative studies the spectra were analyzed by the Grams 386 program. DSC thermograms were obtained by a Shimadzu DSC 60 system previously calibrated with indium. An amount of 8–10 mg polymer was packed in aluminum DSC pans before being placed in a DSC cell and heated under nitrogen gas from 30 to 200 °C at a heating rate of 20 °C·min^−1^. The data were collected from the second scan for all samples. No degradation of PCL, E-VAL, and their blends was observed from DSC thermograms. Note that *T_g_* was taken as the midpoint of the heat capacity change with temperature and T_m_ at the top of the melting changed with temperature.

#### 3.4.2. Mechanic Properties

The effect of the incorporation of E-VAL in the PCL matrix on its mechanical properties was studied in their wet state, based on ASTM standards, by a ZWICK Z100 using a cross displacement speed of 1 mm∙min. Samples of 4 × 4 × 8 mm^3^ were immersed in an aqueous solution containing 10% Dulbecco’s modified Eagle’s medium (DMEM) enriched in fetal bovine serum (FBS) and then recovered and wiped with tissue paper before examination. The results of the stress–strain tests and the compression modulus, *E*, were evaluated by load displacement measurements, and the elastic zone of the stress–strain curve was obtained.

#### 3.4.3. Surface Wettability

The surface wettability of PCL, E-VAL, and their blends was examined on well-dried 4 × 4 × 8 mm^3^ samples through static contact angle measurements at 10 s using a Theta Lite optical tensiometer (Attension, Biolin Scientific, Finland). The contact angle was measured using One Attension 1.0 type software on a droplet of water deposited on the surface of dry samples. Three measurements were performed for each sample, and the final value was taken from their average calculated from the arithmetic mean.

#### 3.4.4. Water Absorbability

The dried PCL scaffolds, of weight m_1_, were immersed in deionized water for 24 h at 37 °C. The water-saturated scaffolds were weighted, m_2_, and the water absorption, *W_a_* (wt%), was calculated by Equation (6):

(6) $$W_{a}\left(\mathrm{wt}\%\right)=\frac{\left(m_{2}-m_{1}\right)}{m_{1}}\times 100$$

#### 3.4.5. Cell Adhesion and Growth Tests

Cell adhesion and proliferation tests on the surfaces of PCL, E-VAL and E-VAL/PCL blends were carried out using MTT (3-(4,5-dimethylthiazol-2-yl)-2,5-diphenyltetrazolium bromide) assay, as described in the literature [64,65]. Briefly, the adhesion tests were realized on 12-well plates in which one of them was treated by collage (5 mg·mL^−1^) as a positive control of adhesion. Each sample of sterilized polymer material in disk form (15 mm diameter) was placed at the bottom of a well and then exposed to 10 × 10^3^ LoVo cells *in* 1.0 mL 10% FBS-supplemented DMEM medium. They all were then incubated in a 5% CO_2_ humid atmosphere and maintained at 37 °C for 24 h for adhesion tests, and for 24 h and 48 h for cell growth tests. After this step, the material disks populated with *LoVo cells* were cultured in the presence of 1% (*v*/*v*) MTT solution (5.0 mg·mL^−1^) for 24 h and 48 h. At the end of the incubation, the test materials were washed two times with sterile phosphate buffer saline (PBS) to remove nonadhered cells; then, 500 µL 0.04 N isopropanol solution was added to each well. The dissolved material was then transferred from each well to another 96-well flat-bottom plate and measured at 550 nm using an ELISA reader (Model 680, BioRad Laboratories, Mississauga, ON, Canada). The percentage of adhesion was evaluated from Equation (7)

(7) $$Adhesion\;\left(\%\right)=\frac{DO_{s}-DO_{nc}}{DO_{pc}-DO_{nc}}\;\times \;100$$

where $DO_{s}$, $DO_{pc}$ and $DO_{nc}$ are the optical densities of the test sample, positive control, and negative control, respectively.

Surface morphology analysis of PCL, E-VAL and E-VAL/PCL dried films coated with gold grids were performed on an SEM (JEOL JSM 6360, Tokyo, Japan) at an accelerating voltage of 15 kV. The samples were first sputter-coated with a thin layer of gold and then observed at a magnification range of 300–3000×. The X-ray diffractions of PCL homopolymer, E-VAL copolymer, and their blends were recorded by a Rigaku D_max_ 2000 X-ray diffractometer using a Cu anode tube at a voltage of 40 kV and a generator current of 100 mA. The diffraction angle range was 0–80 2θ. The samples were used as thin films, whereas pure naphthalene was analyzed as powder.

#### 3.4.6. Porosity Evaluation

After confirming the absence of residual naphthalene incrusted within the scaffold pores by proton nuclear magnetic resonance (^1^HNMR) from the absence of signals at 7.4 and 7.8 ppm, the evaluation of the porosity, *V_f_*, was performed through density measurement. Thus, dried scaffolds (*m*_1_) were immersed in ethanol until saturation ($m_{\infty }$), and the porosity was calculated from the following equation:

(8) $$V_{f}\left(wt\%\right)=\frac{\left(m_{\infty }-m_{1}\right)}{(m_{\infty }-m_{1})d_{1}+m_{1}d_{2}}\times 100$$

where *d*_1_ and *d*_2_ are the densities of polymeric material and ethanol, respectively.

## 4. Conclusions

We demonstrated the preparation of a new material by blending two vital polymers, namely, PCL and E-VAL to enhance the properties of PCL for use in tissue engineering applications. The miscibility of the polymer blends was proved by DSC and confirmed by FTIR analysis. The effect of E-VAL incorporated in the PCL matrix on the mechanical performance of the fabricated scaffold revealed that the values of the compressive modulus and maximum stress of wet PCL, E-VAL, and E-VAL/PCL scaffolds dropped practically by half compared with that of pure PCL when the E-VAL incorporated in the blend goes from zero to 100% by weight. Loading the E-VAL in the PCL matrix enhances the mechanical properties of the scaffold. The wettability of E-VAL/PCL materials increased significantly. The cell adhesion tests on the prepared polymeric materials revealed a significant improvement in LoVo cell adhesion on PCL when E-VAL was incorporated in the PCL. For example, only 10 wt% of E-VAL increased the adhesion performance from 62.18 to 183.32%. The specific cell growth during 24 h and 48 h culture periods on the PCL, E-VAL, and blends showed the highest growth rate on E-VAL/PCL50 compared with the other tested specimens. The SEM micrographs of the cross section of scaffolds reveal spongy form with interconnected pores. The effects of the porogen microparticle diameters on the pore diameters and porosities of the prepared scaffolds revealed that the size of the pores formed is much smaller than expected. Although these pores were smaller than the size of the porogen, the internal pore surfaces appeared sufficiently large to allow cells to proliferate.

## Figures and Tables

**Table 1:** Fractions of associated and free carbonyl bands in 1700–1800 cm^−1^ in the E-VAL/PCL blends.

Blend	Carbonyl Associated		Carbonyl Free		*F*_1_
	*A_a_* (unit^2^)	*ν* (cm^−1^)	*A_na_* (unit^2^)	*ν* (cm^−1^)	
10:90	13.8	1736	77.0	1770	0.16
25:75	24.4	1737	61.6	1754	0.30
50:50	37.6	1737	68.0	1756	0.38
75:25	16.8	1737	88.0	1754	0.17
90:10	44.0	1738	48.0	1755	0.50

**Table 2:** Comparison of the glass transition temperatures of the E-VAL/PCL blend with different compositions determined by experiment, calculation, and using the Fox equation and Gordon–Taylor equation.

System E-VAL/PCL	$T_{g}^{Exp}$ (°C)	$T_{g}^{Cal}\;(\mathrm{Blend})$ (°C)	$T_{g}^{Fox}$ (°C)	$T_{g}^{G–T}$ (°C)
(100:0)	58	-	-	-
(90:10)	35	58.2	40.5	41.1
(75:25)	16	28.5	18.0	18.7
(50:50)	−14	−1.0	−14.0	−12.8
(25:75)	−43	−30.5	−39.0	−38.5
(10:90)	−53	−48.2	−52.1	−52.0
(0:100)	−60	-	-	-

**Table 3:** Comparative data of the melting temperatures of E-VAL/PCL blends with different compositions.

System E-VAL/PCL	PCL		E-VAL	
	*T_m_* (°C)	Δ*H_m_* (J·g^−1^)	*T_m_* (°C)	Δ*H_m_* (J·g^−1^)
(100:0)	-	-	183	73.70
(90:10)	52	8.34	180	47.83
(75:25)	56	14.18	178	31.32
(50:50)	67	45.46	177	27.02
(25:75)	65	69.19	175	24.57
(10:90)	64	73.12	167	19.65
(0:100)	62	75.10	-	-

Δ*H_m_* = 72.0 J∙g^−1^ for E-VAL 27% [45].

**Table 4:** Compressive mechanical properties of wet scaffolds.

Scaffold	E (MPa)	σ (MPa)
PCL	66.67 ± 1.23	11.06 ± 1.11
E-VAL/PCL90	60.05 ± 1.37	10.25 ± 1.08
E-VAL/PCL75	54.32 ± 1.33	9.06 ± 1.04
E-VAL/PCL50	50.43 ± 1.20	8.03 ± 0.85
E-VAL/PCL25	46.15 ± 1.43	7.00 ± 0.64
E-VAL/PCL10	42.86 ± 1.20	5.90 ± 0.61
E-VAL	37.50 ± 1.56	5.25 ± 0.44

**Table 5:** Pore diameters and porosity of pure PCL, E-VAL and E-VAL/PCL scaffolds.

System	Average Porogen Diameter (μm)	Pore Diameter (μm)	Porosity (%)
PCL	120–190	40–150	82.35
E-VAL/PCL10	120–190	57–165	85.12
E-VAL/PCL25	120–190	60–130	86.83
E-VAL/PCL50	120–190	73–140	88.03
E-VAL/PCL75	120–190	56–130	82.22
E-VAL/PCL90	120–190	53–114	80.32
E-VAL	120–190	50–80	75.12

**Table 6:** Preparation of PCL/E-VAL blends in dimethylformamide

Sample	E-VAL/PCL (wt%)	E-VAL (g)	PCL (g)
PCL	100:0	0	10.0
E-VAL/PCL90	90:10	1.0	9.0
E-VAL/PCL75	75:25	2.5	7.5
E-VAL/PCL50	50:50	5.0	5.0
E-VAL/PCL25	25:75	7.5	2.5
E-VAL/PCL10	10:90	9.0	1.0
E-VAL	0:100	10	0
